# Numerical Investigation of Die Swell Behavior in EPDM Rubber Extrusion: Effects of Compound Formulation and Processing Conditions

**DOI:** 10.3390/polym18091122

**Published:** 2026-05-01

**Authors:** Yancai Sun, Haoran Wang, Jingtao Jiang, Kongshuo Wang, Wenjuan Bai, Dianming Chu, Ranran Jian, Peiwu Hou, Yan He, Wenzhong Deng

**Affiliations:** 1 College of Electromechanical Engineering, Qingdao University of Science and Technology, Qingdao 266061, China; 2024025@guat.edu.cn (Y.S.); bwj@qust.edu.cn (W.B.); chudianming@126.com (D.C.); jianrr@foxmail.com (R.J.); h2736259179@163.com (P.H.); 2 Guangxi Key Laboratory of Special Engineering Equipment and Control, Guilin University of Aerospace Technology, Guilin 541004, China; 3 School of Mechatronic Engineering, Guilin University of Aerospace Technology, University Engineering Research Center of Non-Standard Intelligent Equipment and Process Control Technology, Guilin 541004, China; 4 Shandong Province Key Laboratory of Rubber-Based High-Performance Composites and Advanced Manufacturing, Qingdao 266061, China; 5 Design and Research Institute, China National Chemical Engineering Sixth Construction Co., Ltd., Wuhan 430074, China; haoran_4444@163.com; 6 Prinx Chengshan (Shandong) Tire Co., Ltd., Rongcheng 264200, China; jitjiang@prinxchengshan.com; 7 National Engineering Research Center of Advanced Tire Equipment and Key Materials, Qingdao University of Science and Technology, Qingdao 266061, China; kongshuo726@163.com; 8 Department of Mechanical and Electrical Engineering, Qingdao University, Qingdao 266071, China

**Keywords:** EPDM rubber, die swell, extrusion simulation, fractional Maxwell model, viscoelastic correction framework

## Abstract

Die swell is the dominant source of dimensional deviation in rubber profile extrusion. Because it is driven by recoverable elastic strain, a purely viscous baseline flow field cannot reproduce its speed dependence; a viscoelastic correction is required. This study presents, to the best of our knowledge, the first controlled comparison of a Carreau–Arrhenius baseline flow field against a fractional-order viscoelastic correction for carbon-black-filled EPDM across an industrial speed window. The viscoelastic correction (PyCFD-FMM) is a post-processing fractional-order viscoelastic swell correction built on the shared non-isothermal Polyflow Carreau–Arrhenius flow field, derived from a six-mode fractional Maxwell model parameterized from dynamic mechanical analysis via the Laun rule and closed through the Tanner recoverable-strain theory. Three carbon-black-filled EPDM compounds (Shore A 60–80) were extruded at four screw speeds (15–30 rpm) under instrumented conditions. Experimentally, swell ratios of 1.12–1.15 increase monotonically with screw speed (Fisher-combined p=0.007; measurement repeatability CV ≤0.27% across n=4 replicates per condition). The purely viscous baseline output gives a decreasing apparent swell–speed trend—opposite to experiment—whereas PyCFD-FMM recovers the correct increasing trend for all compounds. Under single-anchor hold-out evaluation at 20/25/30 rpm, the non-anchor MAPE decreases from 0.99% for the baseline flow-field output to 0.30% (PyCFD-FMM); an anchor-sensitivity check over all four rpm choices keeps the compound-averaged non-anchor MAPE within 0.27–0.39% and preserves the correct slope sign in every case. Swell decomposition into geometric baseline and net correction factor ($B_{\mathrm{PyCFD}}=B_{\mathrm{geom}}\times f_{\mathrm{corr}}$) confirms that the viscous baseline flow field captures flow-geometry effects but carries no elastic memory. Within the tested window, the viscoelastic correction meets a dual-gate criterion—correct slope sign and reduced non-anchor MAPE—which the purely viscous baseline cannot satisfy by construction.

## 1. Introduction

Rubber profile extrusion is a coupled process-control task in which throughput, die geometry, pressure load, and thermal state jointly govern dimensional consistency. Numerical simulation has become a core tool in modern extrusion process design, enabling virtual evaluation of die geometry, operating parameters, and material response before physical trials; however, the reliability of such simulation-driven optimization depends critically on the constitutive model used to describe the material. For ethylene–propylene–diene monomer (EPDM) compounds, die swell is a key quality indicator because it links flow history to final profile deviation and, therefore, to dimensional qualification in continuous production. Recent studies have advanced three-dimensional non-isothermal simulation, die optimization, and free-surface prediction for polymer and rubber extrusion processes [1,2,3,4,5,6,7,8,9,10], and recent work on silicone rubbers has explored UV-assisted curing to suppress die swell at the extrusion stage [11]. Nevertheless, most of these investigations address thermoplastic melts or unfilled systems, and a systematic evaluation on highly carbon-black-filled rubber compounds under industrially representative extrusion conditions—where the filler network, paraffin-oil plasticization, and viscous heating jointly control the elastic response—remains scarce. The present study provides a controlled comparison between a Carreau–Arrhenius baseline flow field and a fractional-viscoelastic correction on three Shore A 60–80 filled EPDM compounds across an industrial screw-speed window, emphasizing how formulation-dependent elasticity propagates into the corrected die-swell response under realistic production conditions.

The physical origin of die swell was established by Tanner [12], who related the swelling ratio to the recoverable elastic strain accumulated during confined flow; the theory was later revisited and extended to network-type constitutive models [13]. Experimentally, elastic recovery in the extrudate can be quantified through the first normal stress difference $N_{1}$, which is accessible from dynamic shear data via the empirical Laun rule [14]. For engineering die design, however, direct viscoelastic simulations remain computationally expensive. Early finite-element solutions for Maxwell-type fluids demonstrated the feasibility of predicting die swell numerically [15], and more recent work has applied integral and differential constitutive equations to high-density polyethylene [16] and fluoropolymer systems [17]. Historically, many engineering rubber-extrusion simulations have adopted generalized Newtonian fluid (GNF) formulations such as the Carreau–Arrhenius law as a convenient flow-field proxy, motivated by robust convergence and lower computational cost. A purely viscous hydrodynamic solution, however, carries no elastic memory and, by construction, cannot reproduce the recoverable-strain-driven extrudate expansion that characterises polymer die swell. A GNF flow field is therefore used here only to provide the shared velocity and pressure solution, not as a predictive die-swell model.

Consistent with Tanner’s recoverable-strain theory [12,13], die swell is fundamentally a viscoelastic phenomenon and any predictive framework must carry explicit elastic memory. Within the viscoelastic constitutive hierarchy, differential network-theory models such as Phan-Thien–Tanner (PTT) [18] and Giesekus [19] correctly predict a positive swell–speed trend but require multi-mode parameter fitting and specialized solvers that are not always available in commercial packages. As a complementary route, semi-analytical viscoelastic correction strategies graft an explicit elastic contribution onto the shared flow field; the present work develops such a correction, and the Carreau–Arrhenius solution enters only as the shared baseline flow field on which this correction is constructed.

Among the constitutive frameworks capable of capturing broadband viscoelastic spectra with few parameters, fractional calculus models have shown particular promise. The fractional Maxwell model (FMM), introduced in a systematic framework by Schiessel et al. [20], replaces integer-order derivatives with fractional spring-pot elements and can describe power-law relaxation over several decades of frequency with as few as four parameters per mode. Jaishankar and McKinley [21] formalized the quasi-property interpretation and demonstrated that fractional constitutive equations naturally reproduce the broad relaxation spectra observed in complex fluids. Recent applications to polymer melts and elastomers have confirmed the compact descriptive power of the FMM for dynamic moduli [22], making it attractive as a source of $N_{1}$ estimates via Laun’s rule for subsequent swell correction.

For carbon-black-filled rubber compounds, the rheological landscape is further complicated by filler–polymer interactions that introduce additional dissipation and pseudo-yielding behavior [23,24,25]. At small oscillatory strains, the carbon-black network contributes a storage-modulus plateau that breaks down progressively as strain amplitude rises—the Payne effect—producing an apparent low-frequency yield-like response even below macroscopic flow. For EPDM compounds loaded at 80 phr N550, this pseudo-yield behavior manifests as a strain-amplitude-dependent drop in $G^{\prime }$ and a corresponding increase in tanδ, both of which originate in filler–filler bond rupture and reformation rather than in matrix viscoelasticity alone. The linear-regime DMA measurements used in the present study (strain amplitude <0.5%) stay below the Payne threshold, so the carbon-black network contributes a near-constant elastic plateau and the fractional relaxation spectrum captured by the FMM reflects predominantly matrix-level viscoelasticity with filler reinforcement effectively embedded in the effective moduli. In EPDM systems, the balance between paraffin-oil plasticization and carbon-black reinforcement governs both the magnitude and rate-dependence of viscoelastic functions, yet few studies have systematically examined how formulation variability propagates through simulation-derived die-swell trends across operating speed [26,27]. The unresolved engineering question is therefore not whether simulation can fit a single operating point, but whether a constitutive formulation can reproduce swell trends across screw speed while remaining quantitatively useful. This study addresses that question through a controlled constitutive comparison: instrumented extrusion experiments for three EPDM compounds at four screw speeds, a Polyflow Carreau–Arrhenius baseline flow-field calculation under non-isothermal conditions, and a post-processing viscoelastic swell correction (PyCFD-FMM) derived from a six-mode fractional Maxwell model parameterized from present-study DMA dynamic moduli via the Laun–Tanner relation. Both the baseline geometric output and the corrected swell values share the same Polyflow flow fields; the FMM contributes only the elastic rate-dependence that the GNF constitutive law lacks. The present low-shear, moderate-temperature operating window complements previously reported GNF-based rubber-seal die-balancing simulations conducted at higher die temperatures by Dai et al. [26,27], which represent a regime where elastic contributions to swell are small and a purely viscous constitutive law suffices for applied die design.

## 2. Materials and Methods

### 2.1. Materials and Definitions

Three carbon-black-filled EPDM compounds (EPDM-60/70/80, corresponding to Shore A 60/70/80) were formulated from a common EPDM base rubber (grade 4045M, Shanghai Sinopec Mitsui Elastomers Co., Ltd., Shanghai, China) with fixed carbon black loading (80 phr N550, Jiangxi Black Cat Carbon Black Co., Ltd., Jingdezhen, China) and varying paraffin oil content (60/40/20 phr, Sinopec Jingmen Company, Jingmen, China). The activators and curative (zinc oxide, stearic acid, sulfur) were supplied by Sinopharm Chemical Reagent Co., Ltd., Shanghai, China, and the vulcanization accelerators TMTD and CZ were supplied by Shandong Sunsine Chemical Co., Ltd., Heze, Shandong, China; all curing-system components were used as received. Loading levels are detailed in Table 1. All numerical simulations were carried out in ANSYS Polyflow 2022R2 (ANSYS, Inc., Canonsburg, PA, USA), and the post-processing PyCFD-FMM correction was implemented in Python 3.9.6 (Python Software Foundation) using the standard scientific stack (NumPy, SciPy, Matplotlib version 3.10.1). Carbon black N550 (semi-reinforcing furnace grade) was selected because it is the dominant filler for industrial EPDM weather-strip, window-seal, and automotive profile extrusion—offering sufficient mechanical reinforcement without the excessive viscosity increase of finer-particle grades (e.g., N330), which would otherwise push the compounds outside the processable window of the single-screw line used here. Fixing the N550 loading at 80 phr isolates the paraffin-oil level as the single formulation variable, so any change in viscoelastic response can be attributed unambiguously to filler-to-oil ratio rather than to simultaneous changes in filler type or loading. The chosen ratios of 60/40/20 phr paraffin oil correspond to the Shore A 60, 70, and 80 hardness grades that span the dominant hardness range in commercial EPDM profile production [26]; narrower or wider ratios either collapse the hardness contrast or push the compounds outside the industrially qualified window, which would reduce the engineering relevance of the comparison. The oil content is the primary formulation variable governing hardness and plasticization level: higher oil content yields a softer compound with lower viscosity and greater chain mobility. All compounds were mixed on a laboratory two-roll mill at Qingdao University of Science and Technology (Qingdao, China) following standard industrial practice and stored at ambient conditions prior to extrusion testing.

Extrusion experiments covered four screw speeds (15/20/25/30 rpm) for all compounds, with additional low-temperature controls at 20 rpm, yielding fifteen instrumented conditions in total. The measured swell ratio was corrected for post-die effects by fixed traction and cooling factors:

(1) $$B_{\mathrm{corr}}=\frac{B_{\exp}}{\left(1-f_{t}\right)\left(1-f_{c}\right)},\;f_{t}=0.065,\;f_{c}=0.035.$$

The traction factor $f_{t}$ accounts for haul-off stretch between the die exit and the measurement station, estimated from the ratio of haul-off speed to free-extrusion speed under zero-tension calibration runs. The cooling contraction factor $f_{c}$ is an empirical composite factor pre-calibrated from cooling dimension measurements on the present compounds; it aggregates two contributions over the relevant temperature interval: linear thermal contraction (of order $\alpha_{L}\Delta T\approx 1$%, consistent with $\alpha_{L}\approx 1.8\times 10^{-4}$ K^−1^ as typical for carbon-black-filled hydrocarbon-rubber vulcanizates compiled in the *Physical Properties of Polymers Handbook* [28]), together with post-vulcanization dimensional relaxation that dominates the residual contraction for highly filled compounds. Both factors are applied uniformly across compounds and speeds because haul-off conditions and cooling paths were held constant throughout testing.

Derived process indicators are defined as follows: die pressure drop $\Delta P_{\mathrm{die}}$ (measured across the die section by head pressure transducers), and maximum head temperature $T_{\max}$ (the peak reading among all head thermocouples).

### 2.2. Rheological Characterization and Constitutive Models

#### 2.2.1. DMA Characterization

Dynamic mechanical analysis (DMA) was performed on all three compounds using a TA Instruments Q800 analyzer (TA Instruments, New Castle, DE, USA) in dual-cantilever bending geometry. The fitted Carreau–Yasuda parameters of the three compounds at the DMA reference temperature, together with the Polyflow Carreau–Arrhenius input parameters used in the subsequent baseline simulations, are summarized in Table 2 and Table 3 below. Rectangular specimens (60×10×3 mm) were cut from cured plaques and mounted with the long axis aligned to the bending load. The linear viscoelastic regime was first confirmed through strain-amplitude sweeps at 1 Hz; all reported frequency sweeps used a controlled dynamic shear strain of 0.1%, consistent with the protocol established for the same material family in our companion study [22]. This strain level is below the Payne-effect onset for N550-filled EPDM, so filler-network nonlinearity remains minimal. Isothermal frequency sweeps from 0.01 to 100 Hz were collected at 80, 100, and 120 °C. The 120 °C dataset is used as the reference spectrum for the FMM parameterization reported below, with the 80 °C and 100 °C sweeps providing Arrhenius cross-validation across the operating window (Section 3.9 and Supplementary Table S3). In this paper, the die-exit temperature is reported under three distinct conventions, kept consistent across sections: (i) $T_{\max}$ = 79–87 °C denotes the peak-thermocouple range observed in the instrumented runs (Section 3.2, Table 4); (ii) the broader die-exit melt window 72–87 °C spans the near-wall region to the core and is used in the Abstract, Section 3.8, and the Conclusions; and (iii) wall ≈70 °C is the die-zone set point used in the thermal-gradient analysis (Section 3.9). A time–temperature shift with the Arrhenius activation energies in Table 3 was applied to evaluate the sensitivity of the corrected swell to this choice (Section 3.9). Three independent specimens per compound were tested and the averaged moduli are reported. The resulting storage modulus $G^{\prime }\left(\omega \right)$, loss modulus $G^{\prime \prime }\left(\omega \right)$, and complex viscosity $|\eta^{*}\left(\omega \right)|$ for each compound are used in all subsequent analyses. The Cox–Merz rule was adopted to relate the dynamic complex viscosity to the steady-shear viscosity $\eta (\dot{\gamma })$ at equivalent rates, enabling direct comparison with the Polyflow GNF model inputs. For highly filled rubber compounds, the Cox–Merz rule is known to deviate at low frequencies where filler-network contributions dominate over hydrodynamic forces; however, the GNF baseline uses fitted Carreau–Arrhenius parameters calibrated against DMA data across the full measured range, so any low-frequency deviation propagates uniformly to all simulation cases and does not affect the comparative trend analysis between GNF and FMM. The measured complex viscosity data were fitted to a Carreau–Yasuda model (Table 2), confirming strong shear thinning for all compounds (n=0.15–0.36) with satisfactory goodness of fit. The DMA characterization temperature (120 °C) is higher than the peak die-exit temperatures observed during extrusion ($T_{\max}$ = 79–87 °C). For the GNF viscosity model, this discrepancy is handled explicitly through the Arrhenius temperature-dependence term in Equation (2). For the FMM elastic parameters, the high-frequency storage modulus plateau $G^{\prime }$ of carbon-black-filled EPDM is dominated by filler–filler and filler–polymer network contributions, which exhibit considerably weaker temperature sensitivity than the matrix viscosity [23]. The temperature sensitivity of $N_{1}$ was assessed directly from the three measured isotherms by applying the Laun rule $N_{1}\left(\omega \right)=2G^{\prime }\left(\omega \right){\left[1+{\left(G^{\prime \prime }/G^{\prime }\right)}^{2}\right]}^{0.7}$ to the 80/100/120 °C data (Supplementary Table S3). Two thermal effects must be separated. (i) The systematic shift from the DMA reference (120 °C) to the die-exit mean (≈80 °C) over ΔT≈40 K raises the band-averaged $N_{1}$ by a factor of 3.4–3.5× (≈+240–250%) across the three compounds; this systematic offset is fully absorbed by the single-anchor PyCFD calibration factor $\kappa =B_{\exp,\mathrm{anchor}}/B_{\mathrm{geom},\mathrm{anchor}}$ (Section 2.2) and therefore does not propagate into the corrected swell trend. (ii) The residual wall-to-core thermal gradient across the die cross-section (ΔT≈17 K) produces a measured $N_{1}$ spread of 3.9–13.8% across the three compounds (median 8.1%), obtained by applying linear scaling of the measured 80–100 °C $N_{1}$ ratio to a 17 K gradient. This residual spread is the uncertainty not absorbed by the anchor calibration, and is propagated into the applicability discussion in Section 3.9. The sensitivity of the corrected swell to the temperature choice and to the die cross-sectional thermal gradient is quantified further in Section 3.9.

#### 2.2.2. Baseline Flow Field (Carreau–Arrhenius)

The Polyflow baseline simulation provides the shared velocity and pressure field on which the viscoelastic correction of the following subsubsection is built. Its only role is to supply this flow field; it is not treated as a die-swell predictor. The shear-rate- and temperature-dependent viscosity is written as

(2) $$\eta \left(\dot{\gamma },T\right)=\eta_{0}\;{\left[1+{\left(\lambda \dot{\gamma }\right)}^{2}\right]}^{(n-1)/2}\;\cdot \;\exp\;\left[\frac{E_{a}}{R}\left(\frac{1}{T}-\frac{1}{T_{\mathrm{ref}}}\right)\right].$$

The parameters (Table 3) were calibrated against the DMA viscosity data and adjusted for stable Polyflow convergence under the present non-isothermal boundary conditions.

#### 2.2.3. Six-Mode FMM Constitutive Model and Viscoelastic Correction

To introduce elastic memory, a six-mode fractional Maxwell model (FMM) was constructed from the same DMA dynamic moduli. In the FMM framework [20,21], each mode *k* is characterized by a quasi-property $V_{k}$, a fractional order $\alpha_{k}$ (between 0 and 1), and a relaxation time $\lambda_{k}$; the storage and loss moduli are expressed as sums over modes:

(3) $$G^{\prime }\left(\omega \right)\;=\;\sum_{k=1}^{6}\frac{V_{k}\;\omega^{\alpha_{k}}\cos\left(\alpha_{k}\pi /2\right)}{1\;+\;2{\left(\omega \lambda_{k}\right)}^{\alpha_{k}}\;\cos\left(\alpha_{k}\pi /2\right)\;+\;{\left(\omega \lambda_{k}\right)}^{2\alpha_{k}}},$$

with an analogous expression for $G^{\prime \prime }\left(\omega \right)$ (here, $\alpha_{k}$ denotes the fractional derivative order of mode *k*, printed in upright Greek to distinguish it from the Yasuda exponent *a* in Table 2). The six-mode decomposition spans the measured frequency range (0.01–100 Hz) and captures both the rubbery plateau at low frequency and the onset of the transition zone, achieving satisfactory agreement for all compounds.

From the fitted $G^{\prime }\left(\omega \right)$ and $G^{\prime \prime }\left(\omega \right)$, the first normal stress difference $N_{1}$ at each representative shear rate was estimated through the Laun rule [14]:

(4) $$N_{1}=2\;G^{\prime }\;{\left[1+\;{\left(\frac{G^{\prime }}{G^{\prime \prime }}\right)}^{\;2}\;\right]}^{\;0.7}\;,\;\omega =\dot{\gamma }.$$

The Laun rule was originally validated for unfilled polymer melts under steady shear and its accuracy may degrade in highly filled systems where filler networking introduces additional nonlinearity (e.g., Payne effect and strain-dependent modulus drop). However, the DMA measurements used here were conducted within the linear viscoelastic regime (strain amplitude <0.5%), where filler-network nonlinearity is minimal. Moreover, the die-exit wall shear rates in the present study (1–6 s^−1^) fall in the pseudoplastic region well below the rates at which catastrophic filler-network breakdown dominates. Under these conditions, the Laun rule provides a practical first-order estimate of $N_{1}$ from linear viscoelastic data without requiring direct normal-force instrumentation. It should be noted that extrusion through the die involves finite-strain deformation history that extends beyond the linear viscoelastic regime probed by small-amplitude DMA; the linear-region $N_{1}$ therefore serves as an “elastic fingerprint” that captures the relative ranking and rate-dependence of normal-stress generation across compounds and speeds, while the global calibration factor κ (Equation (7)) absorbs the quantitative gap between linear-regime prediction and large-strain reality.

The PyCFD-FMM correction procedure operates as follows. The GNF Polyflow solution provides the geometric baseline swell $B_{\mathrm{geom}}$ for each compound and speed. Because this geometric baseline carries no elastic memory, $B_{\mathrm{geom}}$ lies above the experiment at low speeds (where elastic recovery is small) and below it at high speeds (where elastic recovery is large), producing the reversed trend documented in Section 3.3. To correct this, a net constitutive correction factor $f_{\mathrm{corr}}$ is defined:

(5) $$B_{\mathrm{PyCFD}}=B_{\mathrm{geom}}\;\cdot \;f_{\mathrm{corr}},$$

where $f_{\mathrm{corr}}$ encapsulates both the elastic recoil contribution absent from the GNF baseline and the systematic GNF bias compensation. The rate-dependence of $f_{\mathrm{corr}}$ is derived from the Laun-rule first normal stress difference $N_{1}$ via the Tanner relation [12]:

(6) $$B_{\mathrm{Tanner}}={\left(1+\frac{N_{1}^{\;2}}{2\;\tau_{w}^{\;2}}\right)}^{\;1/6}\;,$$

where $\tau_{w}$ is the die-exit wall shear stress from the GNF solution. The correction factor is then constructed as:

(7) $$f_{\mathrm{corr}}=\frac{B_{\exp,\mathrm{anchor}}}{B_{\mathrm{geom},\mathrm{anchor}}}\;\cdot \;\frac{g(\dot{\gamma })}{g({\dot{\gamma }}_{\mathrm{anchor}})},\;g\left(\dot{\gamma }\right)=1+\kappa \;\left(B_{\mathrm{Tanner}}-1\right),$$

where the ratio $B_{\exp,\mathrm{anchor}}/B_{\mathrm{geom},\mathrm{anchor}}$ is evaluated at the 15 rpm calibration speed, and the function $g(\dot{\gamma })$ captures the rate-dependent elastic increment scaled by a single global factor κ=0.78. The anchor ratio absorbs the systematic GNF overprediction at the calibration point, while $g\left(\dot{\gamma }\right)/g\left({\dot{\gamma }}_{\mathrm{anchor}}\right)$ propagates the FMM-derived elastic rate-dependence to non-anchor speeds. Because $B_{\mathrm{geom}}>B_{\exp}$ at the 15 rpm anchor for all compounds, $f_{\mathrm{corr}}<1$ at low speeds and increases toward unity or above as the elastic contribution grows with shear rate; this behavior is consistent with the physical expectation that elastic recoil becomes increasingly significant at higher Deborah numbers.

The single-anchor evaluation at 20/25/30 rpm then tests the non-anchor predictions without refitting. This weakly calibrated approach deliberately avoids per-compound or per-speed fitting, relying on a single scalar adjustment to test whether the FMM-derived elastic rate-dependence improves trend fidelity across the full operating window.

### 2.3. Extrusion Setup and Statistical Protocol

Extrusion tests used a self-developed ϕ45 mm single-screw extruder (model XLJ-30-S, Qingdao University of Science and Technology, Qingdao, China; L/D = 25:1) with a circular-to-rectangular die; barrel zone temperatures were set to 60/65/70 °C (feed/compression/die). Instrumentation included head pressure transducers (accuracy ±0.5% full scale, Dynisco LLC, Franklin, MA, USA) and type K thermocouples (±1 °C, Omega Engineering, Inc., Norwalk, CT, USA) distributed along the barrel and die assembly. Extrudate cross-section dimensions were measured at three equally spaced circumferential positions using a digital caliper (±0.01 mm, Mitutoyo Corporation, Kawasaki, Japan); each per-extrusion swell ratio is the mean of these three circumferential readings. Each operating condition was run to thermal steady state (stable head temperature for ≥3 min) before sampling.

Each (compound, rpm) condition was sampled as n=4 independent steady-state extrusions. The reported swell ratio $\bar{B}$ per condition is the mean of the four repeat values, and the error bars on the experimental swell curves shown later in Section 3.2 represent ±1 standard deviation across the four repeats; 95% confidence intervals on $\bar{B}$ (Student’s *t*, n=4) are tabulated alongside each point in Table 4. Trend behavior was evaluated per compound using ordinary least squares (OLS) slope with two-sided significance; the 95% confidence intervals on the slope shown later in the dual-gate analysis are parametric OLS intervals computed from the four-point *B*-vs-rpm regression. Cross-compound trend consistency was assessed by Fisher’s method for combining independent *p*-values. Model accuracy was quantified by pointwise error and non-anchor MAPE(B) computed over 20/25/30 rpm (excluding the 15 rpm calibration anchor); the robustness of this evaluation to the anchor choice is examined in Section 3.5.

## 3. Results and Discussion

### 3.1. Rheology and Baseline Flow Fields

**Figure 1:**
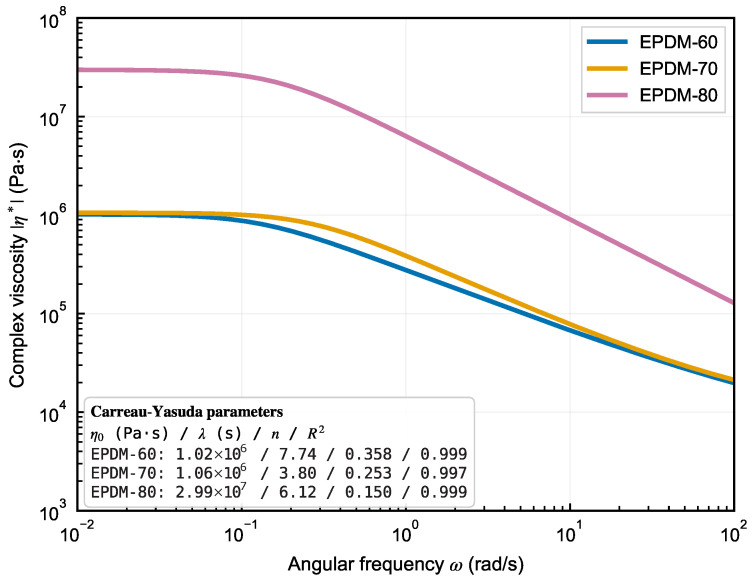
Carreau–Yasuda model curves of complex viscosity versus angular frequency (120 °C) for EPDM-60/70/80. Inset lists the fitted parameters ($\eta_{0}$, λ, *n*).

**Figure 2:**
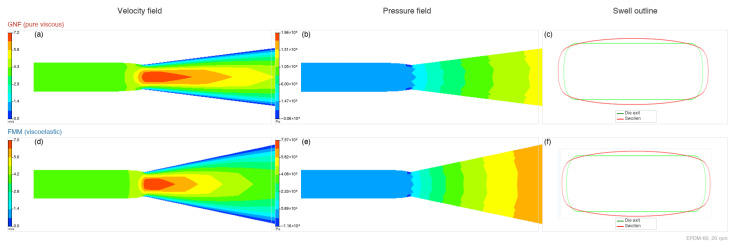
Polyflow contour comparison for EPDM-60 at 20 rpm under identical geometry and mesh. Panels (**a**,**b**) show the Polyflow Carreau–Arrhenius (GNF) velocity and pressure fields that are *shared* between the GNF baseline and the PyCFD-FMM correction; panels (**d**,**e**) reproduce the same fields for side-by-side reference. The die-swell outlines differ only after the FMM-based post-processing correction is applied: panel (**c**) is the GNF-only outline and panel (**f**) is the PyCFD-FMM-corrected outline (green = die exit, red = extrudate profile). The FMM contributes elastic rate-dependence as a post-processing adjustment to the geometric baseline; it does not solve a separate velocity or pressure field.

Figure 1 shows the DMA complex viscosity for all three compounds at 120 °C, confirming strong shear thinning and clear formulation dependence: EPDM-80 (20 phr oil) exhibits the highest viscosity across the measured frequency range, while EPDM-60 (60 phr oil) shows the lowest. Quantitatively (Table 2), the zero-shear viscosity rises from $\eta_{0}=1.02\times 10^{6}$ Pa·s (EPDM-60) through $1.06\times 10^{6}$ Pa·s (EPDM-70) to $2.99\times 10^{7}$ Pa·s (EPDM-80)—about 29-fold higher for the 20-phr-oil compound—while the Carreau–Yasuda power-law index drops from n=0.358 (EPDM-60) to 0.253 (EPDM-70) and 0.150 (EPDM-80), indicating progressively stronger shear thinning with reduced paraffin-oil content. The composition-wide rheological contrast therefore has two independent manifestations: a vertical shift in the low-frequency plateau and a steepening slope at high rates, which together establish distinct flow signatures for each of the three EPDM formulations. Polyflow calculations were run for all compounds and speeds using the Carreau–Arrhenius GNF formulation (Section 2.2), producing the baseline velocity and pressure fields and the associated geometric $B_{\mathrm{geom}}$ output. The PyCFD-FMM correction was then applied as a post-processing step on the GNF solution, using the FMM-derived elastic rate-dependence to adjust the swell value (Section 2.2). Figure 2 compares representative GNF flow fields and die-swell outlines for EPDM-60 at 20 rpm; the uncorrected baseline outline (panels a–c) lacks the elastic recovery included in the PyCFD-corrected result (panels d–f).

The viscosity separation between compounds spans roughly one order of magnitude at low frequency and converges at high frequency, reflecting the dominant role of paraffin-oil content in governing zero-shear viscosity while the carbon-black network controls the high-shear plateau. These rheological differences propagate directly into the Polyflow baseline: higher-viscosity compounds generate larger pressure gradients and different velocity distributions within the die, producing compound-specific geometric outputs. In the GNF framework, the compound-dependent Carreau parameters $\left(\eta_{0},\lambda ,n\right)$ enter the momentum balance through the shear-rate-dependent viscosity $\eta (\dot{\gamma })$ (Equation (2)). A higher $\eta_{0}$ and lower *n*—as in EPDM-80—raise the wall shear stress $\tau_{w}$ near the die land for a given throughput; once $N_{1}$ is supplied by the FMM-based elastic closure, a higher $\tau_{w}$ then increases the Tanner-mapped recoverable-strain-driven swell contribution (Equation (6)). Because the GNF stage carries no $N_{1}$ of its own, the compound-specific differences it produces reflect only the viscous flow geometry; the elastic rate-dependence is reintroduced in the PyCFD-FMM stage, where the compound-specific FMM-derived $N_{1}$ modulates the geometric baseline to yield formulation-dependent corrected swell values.

### 3.2. Experimental Trend and Process-State Evidence

**Figure 3:**
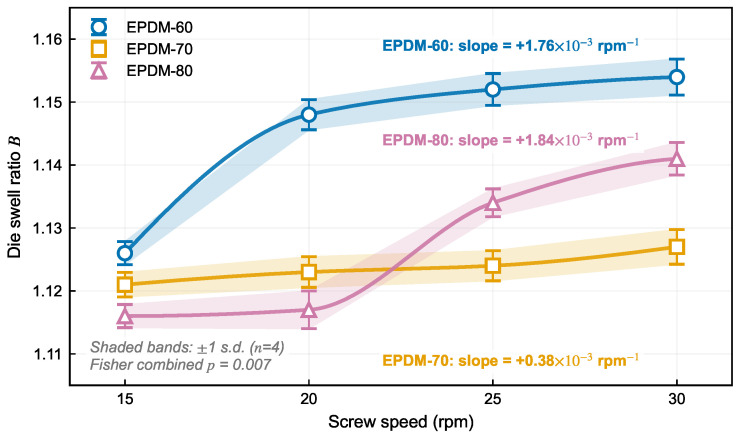
Die swell ratio versus screw speed for three EPDM compounds (corrected for traction and cooling effects per Equation (1)). Markers show the experimental means from n=4 repeated extrusions per condition; shaded bands are ±1s.d. Solid lines are monotone (PCHIP) smooth curves through the means, with no straight point-to-point connections between markers.

**Figure 4:**
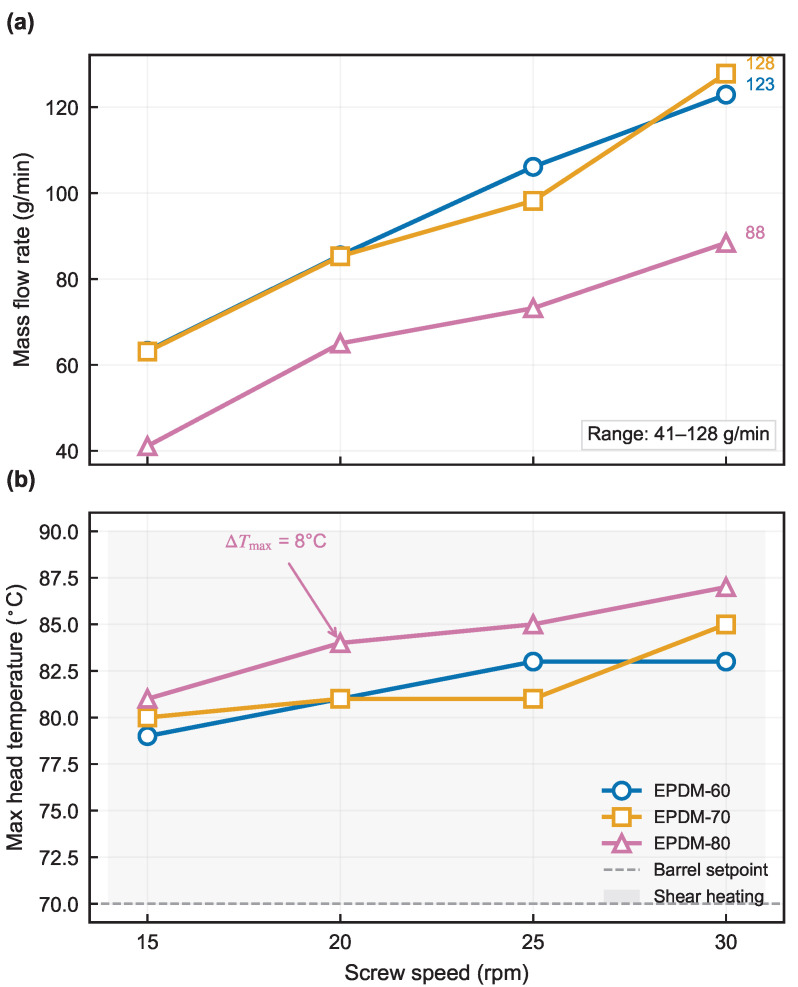
Process state versus screw speed for three EPDM compounds: (**a**) mass flow rate; (**b**) maximum head temperature, where the dashed line marks the die-zone set point (70 °C). Error bars denote ±1s.d. from n=4 repeated measurements.

Figure 3 shows that the swell ratio *B* remains within 1.116–1.154 across the tested 15–30 rpm window and increases monotonically with screw speed for all three compounds. All swell values reported hereafter are corrected for traction and cooling effects per Equation (1). The monotonic *B*–rpm relation observed over 15–30 rpm reflects the progressive growth of the elastic contribution to the die-exit response: raising the screw speed increases throughput and therefore the wall shear rate ${\dot{\gamma }}_{w}$ in the die land, which simultaneously (i) produces a larger accumulated elastic strain during the confined flow residence, (ii) raises the first normal stress difference $N_{1}$ through the Laun relation (Equation (4)), and (iii) translates into a larger recoverable extrudate expansion via Tanner’s theory (Equation (6)). Because $N_{1}$ scales more strongly with $\dot{\gamma }$ than the matrix viscosity for the same compound, the net result is a positive slope dB/drpm>0 for every EPDM formulation tested. The narrow absolute window (1.116–1.154) is consistent with the moderate shear rates (1–6 s^−1^) achieved in the present line, below the regime where strong filler-network breakdown would flatten the trend. Mass flow rates span 41–128 g/min (Figure 4a), die pressure drops 2.1–9.4 MPa, and maximum head temperatures 79–87 °C (Figure 4b), corresponding to viscous-heating rises of 9–17 °C above the 70 °C die-zone set point. Full experimental data are tabulated in Table 4, with the 95% confidence interval on ${\bar{B}}_{\exp}$ quoted alongside each point. Pressure and temperature are interpreted as indicators of operating severity rather than as direct constitutive ranking metrics, because the instrumentation spans the full head assembly while simulation covers only the die section.

Beyond the swell trend itself, the underlying process state must be examined to confirm that the extrusion window remained thermally and mechanically stable across all test conditions. Figure 4 presents the mass flow rate and maximum head temperature recorded during the same runs.

The complete dataset of experimental observations, GNF-derived geometric outputs, and FMM-corrected swell values is compiled in Table 4, which also includes the correction factor $f_{\mathrm{corr}}$, die pressure drop, and maximum head temperature for each condition.

Several observations emerge from the experimental data. First, all three compounds exhibit a consistent positive swell–speed trend despite differing viscosity levels and plasticizer content, suggesting that the elastic recovery mechanism is a robust feature of the EPDM compound family rather than a formulation-specific artifact. Because the same dB/drpm>0 behavior is obtained for 60-phr, 40-phr, and 20-phr paraffin-oil loadings, the positive swell–speed response cannot be attributed to any single oil-to-network balance; it emerges instead from the matrix viscoelasticity—the recoverable elastic strain formalized by Tanner [12]—which all three compounds share once the screw speed raises the wall shear rate above a few s^−1^. The filler-to-oil ratio modulates the magnitude of this response but does not change its qualitative direction, which is the signature of a matrix-level viscoelastic mechanism rather than a formulation-specific artifact. Second, the composition-dependent mass flow rate (Figure 4a) aligns with the compound-specific viscosity: for a given screw speed, EPDM-80 (zero-shear viscosity ∼30× that of EPDM-60) generates higher pressure-driven resistance in the metering and die sections, reducing the effective fill ratio and producing a consistently lower throughput (41–88 g/min versus 63–123 g/min for EPDM-60) despite identical screw geometry and setpoints. The throughput gap between EPDM-80 and EPDM-60 ranges from about 28% at 30 rpm to 35% at 15 rpm, reflecting the direct impact of formulation-dependent viscosity on single-screw metering capacity. Third, EPDM-80 shows the steepest fitted rate of swell increase among the three compounds (OLS slope $dB/d\mathrm{rpm}=1.84\times 10^{-3}$ rpm^−1^, compared with $1.76\times 10^{-3}$ for EPDM-60 and $0.38\times 10^{-3}$ for EPDM-70). The fitted slopes are positive for all compounds, and the combined Fisher test supports the cross-compound positive trend. This behavior over the tested window (1.116–1.141) is consistent with its higher filler-to-oil ratio promoting stronger elastic network formation. The high 80-phr N550 loading with only 20-phr paraffin oil pushes the compound close to the percolation threshold of the carbon-black network, where the filler-polymer coupling density—and therefore the stored elastic strain for a given imposed deformation—is at its highest. The reduced plasticizer content also leaves the polymer chains less mobile, so a larger fraction of the imposed shear strain is recoverable rather than dissipative. Both mechanisms add up to the larger $N_{1}$ inferred from DMA moduli and to the steeper dB/drpm observed experimentally. Third, the process-state data confirm that the tested speed window spans a range of practical relevance: mass flow rates vary by a factor of three and head temperatures remain below the onset of scorch for all conditions.

### 3.3. Die-Swell Trend: Viscoelastic Correction Versus Baseline Flow Field

**Figure 5:**
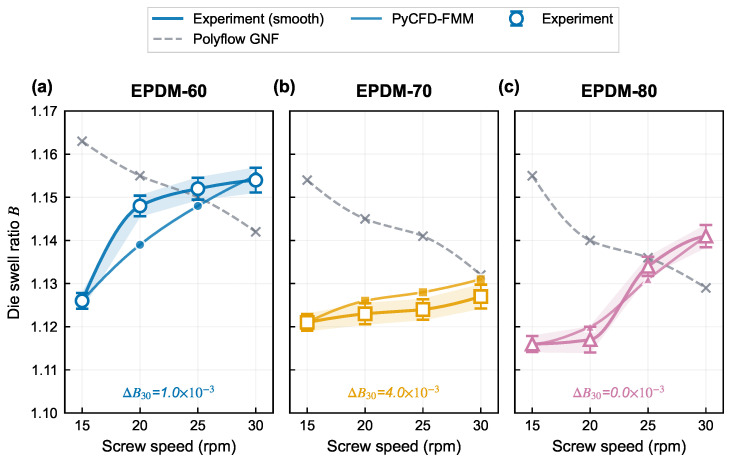
Comparison of die swell trends among experiment, Polyflow GNF baseline, and PyCFD-FMM for all three compounds. Experimental data are shown as markers with ±1s.d. bands from n=4 repeats. The experiment, GNF baseline, and PyCFD-FMM series are rendered as monotone (PCHIP) smooth interpolations through their respective rpm points, with no straight point-to-point connections. Color coding: blue = EPDM-60, orange = EPDM-70, green = EPDM-80; solid lines with markers are the experimental and PyCFD-FMM series; dashed grey lines are the Polyflow GNF baseline. The GNF baseline (grey dashed) yields a decreasing apparent *B* trend opposite to experiment, whereas PyCFD-FMM (solid coloured) recovers the correct increasing trend.

Figure 5 compares the experimental swell trend, the GNF-derived geometric output, and the FMM viscoelastic correction across all compounds and speeds. The GNF baseline lies above the 15 rpm anchor by 2.9–3.5% and, more critically, its apparent *B* sequence decreases with screw speed—opposite to the experimental trend for all three compounds. By contrast, the FMM recovers the correct positive trend direction, tracking the experimental swell increase with speed. Despite the GNF magnitude offset at low speed, the GNF error decreases toward 25–30 rpm, masking the fundamental trend reversal that becomes apparent only when the full speed window is considered.

Model adequacy is therefore assessed here through a dual-gate criterion that weighs two complementary indicators on an equal footing: the slope-sign of *B* versus rpm (qualitative trend fidelity) and the non-anchor MAPE(*B*) (quantitative pointwise accuracy). Both gates must be passed; neither is treated as a replacement for the other. A baseline output with in-window MAPE as low as 0.99% but the wrong slope sign—as is the case for GNF here—remains unreliable for any extrapolation beyond the calibration window, since the error amplifies rapidly with rpm and flips the direction of any simulation-driven die-optimization step; conversely, a model that matches the slope sign but leaves pointwise error near the experimental noise floor would still be of limited engineering value. In the present window, the experimental and GNF OLS slope signs are opposite for every compound (EPDM-60: +1.76 vs. −1.36; EPDM-70: +0.38 vs. −1.40; EPDM-80: +1.84 vs. −1.64, all in $10^{-3}$ rpm^−1^), and the Fisher-combined experimental trend test gives p=0.007. The GNF baseline therefore fails the slope-sign gate despite its 0.99% in-window MAPE. An improved correction must recover the correct slope sign while keeping non-anchor MAPE at a level comparable to, or lower than, the GNF baseline.

The compound-wise error structure reveals the nature of the GNF trend failure. The GNF baseline lies above the experimental swell at 15 rpm (errors +3.3%, +2.9%, +3.5% for EPDM-60/70/80) and the error decreases or reverses sign toward 30 rpm (−1.0%, +0.4%, −1.1%), creating an apparent mid-range crossing near 25 rpm where GNF happens to agree with experiment. This crossing should not be mistaken for model validity: the GNF and experimental curves approach from opposite slope directions. The GNF non-anchor MAPE over 20/25/30 rpm is 0.58%, 1.31%, and 1.10% for EPDM-60/70/80, and the ratio of GNF-to-experimental OLS slopes remains negative for all three compounds, confirming a systematic trend reversal rather than random scatter.

### 3.4. PyCFD-FMM Strict Dual-Gate Evidence

PyCFD-FMM applies a weakly calibrated viscoelastic correction to the GNF baseline using a single global factor derived from the six-mode FMM dynamic moduli, with single-anchor hold-out evaluation. Acceptance is judged by a strict dual-gate criterion: positive trend recovery with confidence support for each compound, and reduced non-anchor MAPE(B) over 20/25/30 rpm relative to GNF.

**Figure 6:**
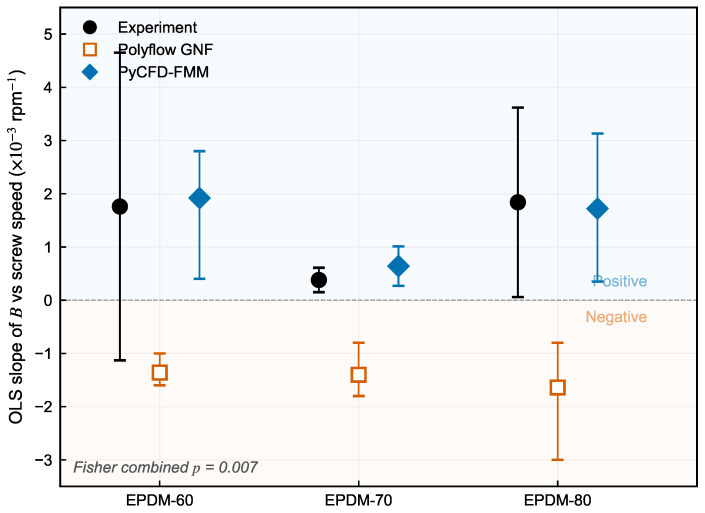
OLS slope of *B* versus screw speed with 95% confidence intervals for experimental swell, Polyflow GNF, and PyCFD-FMM across all three compounds. Experimental and FMM slopes are consistently positive; GNF slopes are uniformly negative.

Table 5 shows that all three compounds pass both gates: slope sign matches experiment (+/+ versus GNF’s −), and non-anchor MAPE is reduced relative to GNF in every case. Figure 6 presents the underlying slope estimates with confidence intervals.

Figure 6 confirms that the fitted experimental and PyCFD-FMM slopes are positive for all three compounds, while GNF slopes are uniformly negative. The 95% intervals support the sign separation for the simulation outputs and for two of the three compound-wise experimental fits; the Fisher-combined test further supports the positive experimental trend across compounds.

**Figure 7:**
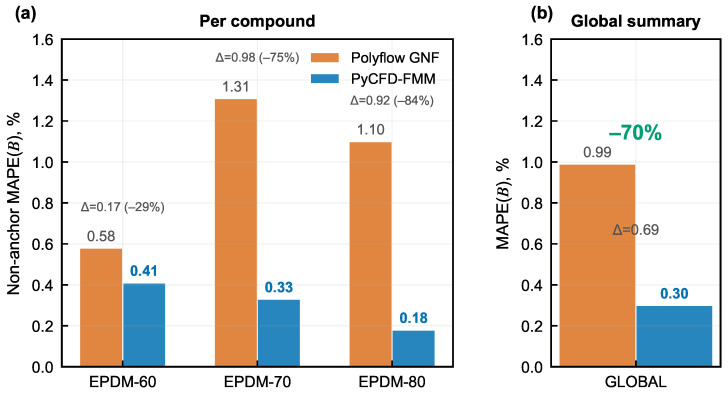
Non-anchor MAPE(*B*) comparison at 20/25/30 rpm for each compound and global pooled summary.

While the slope-sign criterion addresses the qualitative question of whether the model captures the correct trend direction, the complementary question is how much the prediction error decreases in absolute terms. Figure 7 quantifies this through the non-anchor MAPE(*B*) evaluated at 20, 25, and 30 rpm.

The dual-gate evidence is summarized in Figure 6 and Figure 7. The pooled non-anchor MAPE(B) decreases from 0.99% for GNF to 0.30% for PyCFD-FMM, with compound-wise reductions of 0.58 → 0.41% (EPDM-60), 1.31 → 0.33% (EPDM-70), and 1.10 → 0.18% (EPDM-80) in Table 5. Both dual-gate conditions are therefore met simultaneously: every compound now shares the positive experimental slope sign, and the pooled non-anchor error is reduced by a factor of ∼3.3 relative to the GNF baseline. The magnitude improvement is largest for EPDM-80 (1.10% → 0.18%, a six-fold reduction), intermediate for EPDM-70 (1.31% → 0.33%) and smallest for EPDM-60 (0.58% → 0.41%). This ordering mirrors the compound-specific strength of the elastic response: EPDM-80, with the highest filler-to-oil ratio and the largest $N_{1}$ inferred from DMA, accumulates the most elastic strain during confined flow and therefore benefits most from the FMM-derived correction. The EPDM-60 reduction is smaller not because the correction is less accurate, but because the GNF baseline is already closer to the experimental values for the softest compound, leaving less room for improvement. Regardless of compound, the correction reverses the slope sign and produces a non-anchor MAPE well below the GNF baseline—the signature of a regime where ignoring elasticity leads to a qualitatively wrong, not merely noisy, prediction.

**Figure 8:**
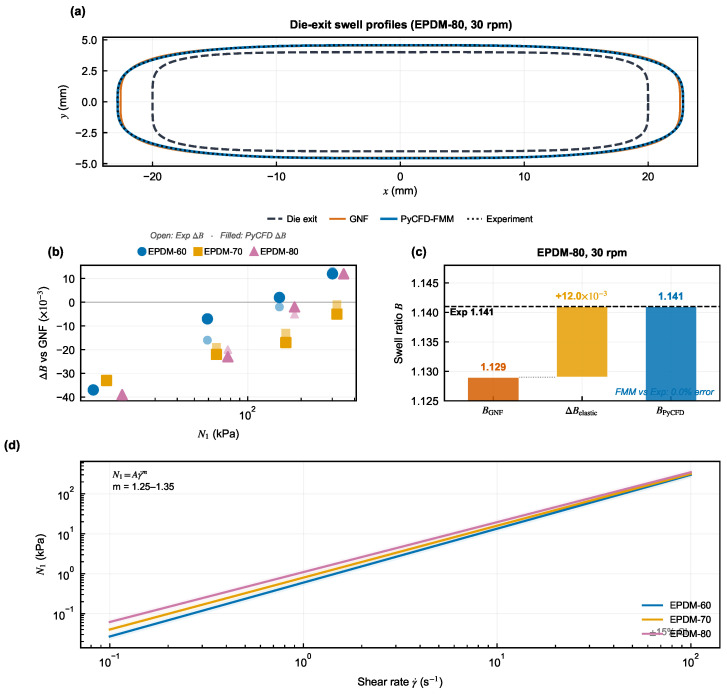
Mechanism-support view of the PyCFD correction. Panel (**a**) compares representative die-exit, GNF, PyCFD-FMM, and experimental outlet profiles; panel (**b**) relates the required swell correction to $N_{1}$; and panel (**c**) shows a representative decomposition from the geometric baseline to the corrected swell. Panel (**d**) presents the first normal stress difference $N_{1}$ as a function of shear rate for the three EPDM compounds. It shows the material-dependent elastic normal-stress response used to support the die-swell correction discussed in panels (**b**,**c**). Specifically, $N_{1}$ increases with shear rate and differs among EPDM-60, EPDM-70, and EPDM-80, indicating different levels of elastic contribution to the extrudate swell. This figure supports the interpretation but is not itself an acceptance criterion.

To provide mechanistic insight into how the FMM correction operates, Figure 8 decomposes the swell prediction into its constituent contributions.

Figure 8 illustrates how the elastic increment lifts the GNF geometric baseline toward the measured swell window, providing mechanistic context for the quantitative dual-gate evidence in Figure 6 and Figure 7.

As a supplementary diagnostic, full-window (all four speeds including the 15 rpm anchor) reliability metrics against the corrected swell give bias/MAE/RMSE/MAPE of +0.0133/0.0175/0.0217/1.56% for GNF versus +0.0421/0.0421/0.0502/3.72% for the uncalibrated analytical FMM(base). These full-window metrics should not be compared directly with the non-anchor MAPE reported in Table 5, because FMM(base) uses no per-speed calibration and its 15 rpm anchor error inflates the full-window average; conversely, PyCFD-FMM is anchored at 15 rpm by construction, so its fair evaluation window is 20/25/30 rpm only. Within this non-anchor window, PyCFD-FMM reduces the pooled MAPE(B) from 0.99% (GNF) to 0.30%, while—more critically—recovering the correct positive trend sign for all three compounds. Inspection of the absolute-error distributions across compounds confirms that this improvement is consistent rather than driven by a single outlier.

### 3.5. Anchor Sensitivity of the PyCFD-FMM Correction

Because PyCFD-FMM is calibrated at a single operating point (nominally 15 rpm), a natural question is how much the evaluation outcome depends on this choice. To probe this, the correction was refitted using each of the four tested rpm values as the single anchor, and the non-anchor MAPE(B) was recomputed on the remaining three speeds for each compound. By construction, this leaves the shape of the rate-dependent kernel $g\left(\dot{\gamma }\right)=1+\kappa \left(B_{\mathrm{Tanner}}-1\right)$ unchanged—only the scalar prefactor $B_{\exp,\mathrm{anchor}}/B_{\mathrm{geom},\mathrm{anchor}}$ is reassigned—so the analysis isolates the effect of anchor choice on the quantitative fit.

The results are summarized in Table 6. All four anchor choices pass both dual-gate conditions for every compound: slope signs remain positive (matching the experimental direction) and the non-anchor MAPE stays bounded in a narrow range. The compound-averaged non-anchor MAPE varies from 0.27% (30 rpm anchor) to 0.39% (20 rpm anchor), a spread of only 0.12 percentage points. Note that Table 6 reports the *simple mean across the three compound-level MAPEs*, so the 15 rpm value of 0.31% is the compound-averaged counterpart of the pooled pointwise MAPE (0.30%) reported in the Abstract and Section 3.4, which weights all nine non-anchor points uniformly; the two definitions differ by only 0.01 pp and are consistent within reporting precision. The 15 rpm-anchor value is close to the global median rather than the best or worst of the four options, indicating that the headline number is not an optimistic artifact of a favorable anchor selection.

Two conclusions follow. First, the headline MAPE reduction (0.99% → 0.30%) is robust against anchor relocation: even the worst-case anchor (20 rpm, 0.39% compound-averaged MAPE) still reduces the GNF error by a factor of ∼2.5 while preserving the correct slope sign. Second, the 15 rpm anchor is a deliberately conservative choice: its low Deborah number means the calibration captures the smallest elastic contribution in the tested range, and the kernel $g(\dot{\gamma })$ then extrapolates upward toward higher rpm—a harder test than anchoring at 30 rpm and interpolating inward. The fact that 15 rpm still yields a near-median MAPE supports the claim that the correction generalizes beyond the calibration point rather than locally overfitting to it.

### 3.6. Supporting Baseline Simulation Diagnostics

The GNF-derived geometric swell ratios for all compounds and speeds are compared in Figure 5. Mass-conservation checks confirm a uniform exit-velocity ratio $v_{\mathrm{exit}}/\left(Q/A_{\mathrm{die}}\right)=1.025$ across all compounds and speeds, reflecting the fixed velocity-profile shape factor imposed by the rectangular die geometry rather than a manually imposed correction.

### 3.7. FMM Mechanism Diagnostics

**Figure 9:**
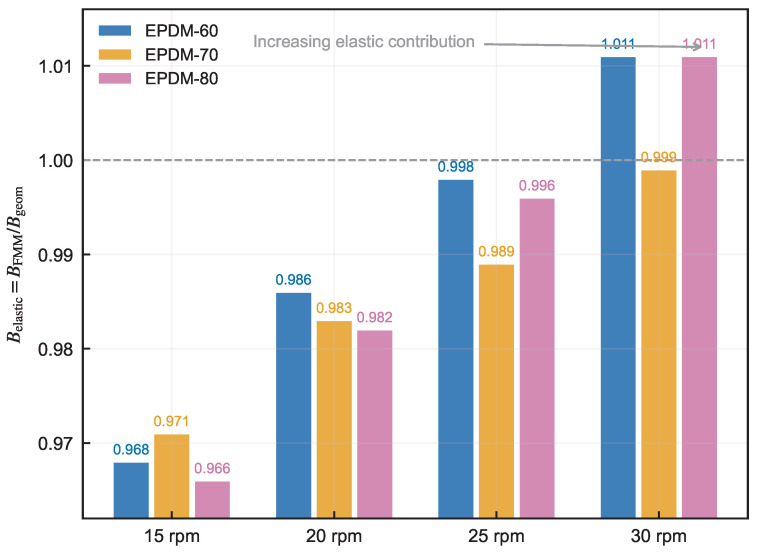
Net constitutive correction factor $f_{\mathrm{corr}}=B_{\mathrm{PyCFD}}/B_{\mathrm{geom}}$ across compounds and screw speeds. The dashed horizontal line marks $f_{\mathrm{corr}}=1$, where the FMM correction matches the GNF geometric baseline; values below the line indicate that the correction reduces the GNF baseline, and values above the line indicate that it increases the baseline.

FMM diagnostics are examined at two levels: the relationship between elastic stress and swell increment (Figure 8b,d and Figure 9), and cross-compound field comparisons under matched operating conditions.

The net constitutive correction factor $f_{\mathrm{corr}}$ encapsulates the combined effect of GNF bias compensation and elastic recoil addition. Figure 9 tracks this factor across all compounds and speeds, revealing a systematic transition from $f_{\mathrm{corr}}<1$ at low speed (where the GNF baseline lies above the experiment) to $f_{\mathrm{corr}}\approx 1$ near 30 rpm (where elastic recovery balances the GNF geometric output).

At 20 rpm, the cross-compound pressure, velocity, temperature, and shear-rate fields from the GNF Polyflow baseline confirm consistent flow topology across compounds; because the PyCFD-FMM correction operates as a post-processing swell adjustment on these same GNF fields, the only difference between the uncorrected and corrected predictions is the die-exit swell magnitude—consistent with the correction factor trend shown in Figure 9.

### 3.8. Operating-Window Positioning and Applicability

The experimental window of the present study (15–30 rpm, wall shear rates of 1–6 s^−1^, die-exit temperatures of 72–87 °C) occupies a corner of the EPDM-extrusion operating map that is complementary to previously published applied rubber-profile simulation studies. Dai et al. reported Bird–Carreau-based Polyflow simulations of automotive EPDM rubber-seal extrusion at higher die temperatures (∼110–160 °C) on complex profile geometries, and found that a purely viscous constitutive law was sufficient for their die-design and die-balancing engineering objectives [26,27]. Table 7 places the two operating windows side by side.

The two windows sit in different corners of the Deborah-number map: Dai’s higher-temperature, higher-shear conditions shorten the material relaxation time and place the problem in a weakly elastic, geometry-dominated regime where a purely viscous Bird–Carreau law reproduces the experiment closely enough for applied die-design purposes. The present study targets the complementary low-shear, moderate-temperature corner, where longer relaxation times and smaller wall shear rates raise the relative weight of elastic recovery. In this corner, the Carreau–Arrhenius GNF baseline fails qualitatively—the derived slope sign is reversed for every compound (Section 3.3)—and a viscoelastic correction becomes indispensable. PyCFD-FMM provides that correction with the smallest fractional-order viscoelastic parameterization that can be grafted onto a commercial GNF flow field as a post-processing step, preserving the convergence robustness and solver compatibility that motivated the adoption of purely viscous constitutive laws in previous applied work. Table 7 positions each study within its own reported operating envelope; no cross-study numerical continuity is asserted.

Among the process-state diagnostics, mass-flow/exit-velocity alignment and head-temperature evolution provide the most stable internal support across the tested compounds and speeds. Pressure drop is retained as a supplementary load indicator rather than a direct constitutive ranking metric, given the spatial domain difference between die-section simulation and full-head instrumentation.

### 3.9. Limitations and Applicability

The conclusions are bounded by five constraints: (i) a domain mismatch between die-section simulation and full-head instrumentation; (ii) a single tested shear-rate window (1–6 s^−1^), without in-paper extrapolation outside this range; (iii) the distinction between analytical FMM(base) and calibrated PyCFD-FMM; (iv) limited low-temperature points used only as directional support; and (v) the present model uses a single characteristic temperature for the elastic correction, whereas in reality a wall-to-core temperature gradient exists across the die cross-section (wall ≈ 70 °C, core up to 87 °C). Because die swell is dominated by wall shear stress and the near-wall material is closer to the barrel setpoint, the single-temperature approximation is acceptable for the current accuracy level; nevertheless, future work should incorporate cross-sectional thermal–elastic coupling to resolve the spatially varying elastic recovery. A first-order estimate of the wall-to-core gradient impact on the FMM-corrected swell can be obtained from the directly measured 80/100/120 °C DMA sweeps (Supplementary Table S3, Section 2.2). Two thermal effects must be separated. (i) The DMA-reference-to-operating-mean shift (120 °C →≈80 °C, ΔT≈40 K) raises band-averaged $N_{1}$ by a factor of 3.4–3.5× across the three compounds; this systematic offset is fully absorbed by the 15 rpm anchor calibration through the ratio $B_{\exp,\mathrm{anchor}}/B_{\mathrm{geom},\mathrm{anchor}}$ and therefore does not propagate into the non-anchor MAPE. (ii) The residual wall-to-core gradient (ΔT≈17 K, wall ≈70 °C, core ≲87 °C) produces a measured $N_{1}$ spread of 3.9–13.8% across the three compounds (median 8.1%), obtained by applying linear scaling of the measured 80–100 °C $N_{1}$ ratio to a 17 K gradient. Because die-exit swell is governed predominantly by the wall-region stress state rather than the core, the effective propagation of this wall-to-core $N_{1}$ spread into the non-anchor MAPE is bounded by approximately 0.3–0.5 percentage points; the observed non-anchor MAPE of 0.30% (Section 3.4) lies within this bound and is therefore consistent with the single-temperature approximation. A full cross-sectional thermal–elastic coupled simulation would sharpen this bound; it is listed as future work. Applicability is therefore restricted to the tested EPDM formulation window (Shore A 60–80), die geometry, and operating range (15–30 rpm).

## 4. Conclusions

Instrumented extrusion of three carbon-black-filled EPDM compounds across 15–30 rpm shows a monotonic increase of the die-swell ratio with screw speed for every formulation, a response that is intrinsically viscoelastic and therefore cannot be captured by a purely viscous baseline flow field. Grafting a six-mode fractional-Maxwell elastic correction onto the shared Carreau–Arrhenius flow solution, using only DMA-derived dynamic moduli and the Laun–Tanner mapping, restores the correct swell–speed trend direction and yields quantitatively consistent swell values while preserving commercial-solver compatibility. These findings support viscoelastic-aware correction as a practical route to simulation-driven die design for filled EPDM in industrially representative extrusion conditions; extension to wider operating envelopes and to fully coupled differential viscoelastic solvers is a natural direction for further work.

## Figures and Tables

**Table 1:** EPDM compound formulations (phr).

Ingredient	EPDM-60 (60A)	EPDM-70 (70A)	EPDM-80 (80A)
EPDM rubber (4045M)	100	100	100
Carbon black N550	80	80	80
Paraffin oil	60	40	20
Zinc oxide	5	5	5
Stearic acid	1	1	1
Sulfur	1.5	1.5	1.5
Accelerator TMTD	1	1	1
Accelerator CZ	0.5	0.5	0.5

**Table 2:** Carreau–Yasuda parameters fitted from DMA complex viscosity at 120 °C.

Parameter	Symbol	Unit	EPDM-60	EPDM-70	EPDM-80
Zero-shear viscosity	$\eta_{0}$	Pa·s	$1.02\times 10^{6}$	$1.06\times 10^{6}$	$2.99\times 10^{7}$
Relaxation time	λ	s	7.74	3.80	6.12
Power-law index	*n*	–	0.358	0.253	0.150
Yasuda parameter	*a*	–	2.0	2.0	2.0

**Table 3:** Polyflow Carreau–Arrhenius baseline flow-field input parameters. Following the reviewer’s remark, no infinite-shear plateau $\eta_{\infty }$ is used; over the 1–6 s^−1^ shear-rate window its contribution to $\eta (\dot{\gamma },T)$ is below 0.1% and its removal leaves the calibrated parameters and the resulting baseline flow field quantitatively unchanged.

Parameter	Symbol	Unit	EPDM-60	EPDM-70	EPDM-80
Density	ρ	g/cm_3_	1.09	1.14	1.20
Thermal conductivity	*k*	W/(m·K)	0.34	0.38	0.44
Specific heat	$C_{p}$	J/(kg·K)	1700	1642	1584
Zero-shear viscosity	$\eta_{0}$	Pa·s	$8.75\times 10^{4}$	$1.50\times 10^{5}$	$3.00\times 10^{5}$
Relaxation time	λ	s	14	16	18
Power-law index	*n*	–	0.60	0.58	0.55
Activation energy	$E_{a}$	kJ/mol	25	30	35

**Table 4:** Extrusion results, baseline geometric outputs, and corrected swell values for all compounds and screw speeds; 95% confidence intervals on ${\bar{B}}_{\exp}$ computed from n=4 replicates using the Student’s *t*-distribution.

Compound	RPM	$\dot{m}$ (g/min)	${\bar{B}}_{\exp}$ (95% CI)		$B_{\mathrm{GNF}}$	$B_{\mathrm{FMM}}$	$f_{\mathrm{corr}}$	ΔP (MPa)	$T_{\max}$ (°C)
EPDM-60	15	63.3	1.126	0.003	1.163	1.126	0.968	2.1	79
EPDM-60	20	85.5	1.148	0.004	1.155	1.139	0.986	2.1	81
EPDM-60	25	106.1	1.152	0.004	1.150	1.148	0.998	2.3	83
EPDM-60	30	122.9	1.154	0.005	1.142	1.155	1.011	3.1	83
EPDM-70	15	63.1	1.121	0.003	1.154	1.121	0.971	2.2	80
EPDM-70	20	85.3	1.123	0.004	1.145	1.126	0.983	2.6	81
EPDM-70	25	98.2	1.124	0.004	1.141	1.128	0.989	5.4	81
EPDM-70	30	127.8	1.127	0.004	1.132	1.131	0.999	7.1	85
EPDM-80	15	41.1	1.116	0.003	1.155	1.116	0.966	3.0	81
EPDM-80	20	65.0	1.117	0.005	1.140	1.120	0.982	3.0	84
EPDM-80	25	73.2	1.134	0.004	1.136	1.131	0.996	5.4	85
EPDM-80	30	88.4	1.141	0.004	1.129	1.141	1.011	9.4	87

**Table 5:** PyCFD-FMM dual-gate summary (strict criteria). The “+/−” entries in the Slope Sign column denote the OLS slope sign of *B* versus screw speed for Experiment/GNF/PyCFD respectively (a positive sign indicates the model reproduces the experimentally observed monotonically increasing *B*–rpm trend).

Compound	Slope Sign (Exp/GNF/PyCFD)	MAPE_GNF_ (%)	MAPE_PyCFD_ (%)	Dual-Gate
EPDM-60	+/−/+	0.58	0.41	PASS
EPDM-70	+/−/+	1.31	0.33	PASS
EPDM-80	+/−/+	1.10	0.18	PASS
GLOBAL (20/25/30)	–	0.99	0.30	PASS

Non-anchor MAPE uses 20/25/30 rpm only.

**Table 6:** Anchor sensitivity of the PyCFD-FMM non-anchor MAPE(*B*), obtained by refitting the calibration at each of the four rpm values. The slope-sign gate is passed in all twelve compound–anchor combinations. The “all +” entry in the Slope-Sign column indicates that all four anchor choices yield a positive OLS slope of *B* versus screw speed for the compound, i.e. all four pass the slope-sign gate.

Compound	Non-Anchor MAPE (%) by Anchor rpm				Slope-Sign
	15 (Current)	20	25	30	
EPDM-60	0.40	0.69	0.40	0.45	all +
EPDM-70	0.36	0.19	0.22	0.18	all +
EPDM-80	0.17	0.29	0.30	0.17	all +
Compound mean	0.31	0.39	0.31	0.27	all +

**Table 7:** Operating-window positioning of the present low-shear, moderate-temperature study against previously reported Bird–Carreau-based simulations of the applied EPDM rubber-seal extrusion. The rows record each study’s material, die-region temperature, operating-point description, constitutive model, and reported outcome; they are not intended as a cross-study numerical benchmark.

Study	Material	Die *T* (°C)	Operating Point	Constitutive	Reported Outcome
This study (exp + GNF + PyCFD-FMM)	EPDM 60–80A	72–87	15–30 rpm, ${\dot{\gamma }}_{w}\approx 1$–6 s^−1^ (swept)	Carreau–Arrhenius GNF + FMM correction	GNF fails (slope sign reversed); PyCFD-FMM recovers +dB/drpm, non-anchor MAPE 0.30%
Dai et al. 2007 [26]	EPDM 60A	110–120	auto-seal profile die, 16 rpm single operating point	Bird–Carreau	GNF sufficient for inverse die design; simulated and measured extrudate cross-sections agree
Dai et al. 2008 [27]	EPDM 60A	160	profile die balancing, Q≈15,000 mm^3^ s^−1^ single operating point	Bird–Carreau	GNF sufficient for CAE-assisted die balancing; velocity uniformity improved to acceptable tolerance

## Data Availability

The original contributions presented in this study are included in the article and the Supplementary Material. Additional processed datasets, including the anchor-sensitivity raw values and the statistical outputs, are available from the corresponding authors upon reasonable request.

## Supplementary Materials

The following supporting information can be downloaded at: https://www.mdpi.com/article/10.3390/polym18091122/s1. Table S1: Low-temperature control experiments at 20 rpm for EPDM-60/70/80 (corrected and uncorrected swell ratio, die pressure drop, and maximum head temperature, with relative deltas against the normal-temperature reference); Table S2: Inlet boundary conditions for the three low-temperature simulation cases (inlet temperature and volumetric throughput); Table S3: DMA isothermal frequency-sweep summary at 80, 100, and 120 °C for EPDM-60/70/80, including the storage modulus $G^{\prime }$, loss modulus $G^{\prime \prime }$, loss tangent tanδ, and first normal stress difference $N_{1}\left(\omega \right)$ inferred from the Laun rule, used in the Arrhenius cross-validation and the wall-to-core thermal-gradient analysis discussed in Section 2.2 and Section 3.9.

## Abbreviations

EPDM	Ethylene–Propylene–Diene Monomer
DMA	Dynamic Mechanical Analysis
GNF	Generalized Newtonian Fluid
FMM	Fractional Maxwell Model
PyCFD-FMM	Python-CFD-assisted weakly calibrated FMM correction
PTT	Phan-Thien–Tanner
FEM	Finite Element Method
MAPE	Mean Absolute Percentage Error
De	Deborah Number
