# Supplementary material for: Numerical Investigation of Die Swell Behavior in EPDM Rubber Extrusion: Effects of Compound Formulation and Processing Conditions
Source: Polymers (Basel). 2026 May 1;18(9):1122. doi: 10.3390/polym18091122 (PMC13165352; doi:10.3390/polym18091122)
Supplement: Supplementary file 1 [file polymers-18-01122-s001.zip › polymers-4238061-supplementary.pdf]

# Supplementary Information
# Numerical Investigation of Die Swell Behavior in EPDM Rubber Extrusion: Effects of Compound Formulation and Processing Conditions

## S1. Low-Temperature Control Experiments at 20 rpm

To provide boundary evidence on thermal sensitivity, low-temperature control experiments were conducted at 20 rpm for all three compounds. The barrel set-point was reduced by approximately 5°C relative to the normal condition.

Table 1: Low-temperature control results at 20 rpm. Correction factors: $f_t = 0.065$, $f_c = 0.035$; $B_{\mathrm{corr}} = B_{\mathrm{exp}}/[(1 - f_t)(1 - f_c)]$; $\Delta P_{\mathrm{die}} = P_3 - P_7$; $T_{\mathrm{max}} = \max(T_1, T_6, T_8, T_{10}, T_{11}, T_{14}, T_{15})$.

| **Compound** | $B_{\mathrm{exp}}$ (normal) | $B_{\mathrm{exp}}$ (low) | $\Delta B_{\mathrm{exp}}$ (%) | $B_{\mathrm{corr}}$ (normal) | $B_{\mathrm{corr}}$ (low) | $\Delta B_{\mathrm{corr}}$ (%) | $\Delta P_{\mathrm{die}}$ (normal) | $\Delta P_{\mathrm{die}}$ (low) | $\Delta\Delta P$ (%) | $T_{\mathrm{max}}$ (normal) | $T_{\mathrm{max}}$ (low) | $\Delta T_{\mathrm{max}}$ (°C) |
|---|---|---|---|---|---|---|---|---|---|---|---|---|
| EPDM-60 | 1.0363 | 1.0338 | −0.24 | 1.1485 | 1.1458 | −0.24 | 2.09 | 2.30 | +10.0 | 81 | 76 | −5 |
| EPDM-70 | 1.0130 | 1.0150 | +0.20 | 1.1227 | 1.1249 | +0.20 | 2.58 | 2.23 | −13.6 | 81 | 76 | −5 |
| EPDM-80 | 1.0083 | 1.0119 | +0.36 | 1.1175 | 1.1215 | +0.36 | 2.98 | 3.63 | +21.8 | 84 | 79 | −5 |

**Interpretation.** At 20 rpm, low-temperature controls show small swell changes (within ±0.36%) and a consistent 5°C decrease in $T_{\mathrm{max}}$. The pressure-drop response remains compound-dependent. EPDM-60 and EPDM-80 show higher $\Delta P_{\mathrm{die}}$ under lower temperature, whereas EPDM-70 shows a lower value. This pattern indicates interaction between temperature, viscosity, and compound-specific flow resistance. Because this control set is sparse (single-speed comparison), it provides directional boundary evidence only and is not used to support standalone mechanistic conclusions in the main text.

## S2. Inlet Boundary Conditions for Low-Temperature Cases

Table 2: Inlet boundary conditions for the three low-temperature simulation cases.

| **Compound / Condition** | $T_{\mathrm{inlet}}$ **(K)** | $Q$ **(m$^3$/s)** |
|---|---|---|
| EPDM-60 / 20 rpm (low $T$) | 345.15 | $1.18 \times 10^{-6}$ |
| EPDM-70 / 20 rpm (low $T$) | 346.15 | $1.24 \times 10^{-6}$ |
| EPDM-80 / 20 rpm (low $T$) | 351.15 | $8.03 \times 10^{-7}$ |

## S3. DMA Isothermal Frequency-Sweep Summary at 80, 100, and 120 °C

To support the Arrhenius cross-validation discussion in Section 2.2.1 and the wall-to-core thermal-gradient analysis in Section 3.9 of the main text, isothermal frequency sweeps were collected on EPDM-60/70/80 at three temperatures (80, 100, 120 °C) using a TA Instruments

Q800 (TA Instruments, New Castle, DE, USA) in dual-cantilever bending at 0.1% strain amplitude. The first normal stress difference $N_1(\omega)$ at each angular frequency $\omega = \dot{\gamma}$ is inferred from the Laun rule:

$$N_1(\omega) = 2\,\mathrm{G}'(\omega)\left[1 + \left(\frac{\mathrm{G}''}{\mathrm{G}'}\right)^2\right]^{0.7}.$$

Table 3 reports the band-averaged moduli, loss tangent, and $\overline{N_1}$ over the measured 0.01–100 Hz window (21 logarithmically spaced points per isotherm). The full point-by-point dataset is archived in the accompanying file `polymers-4238061-S3_DMA_data.csv`.

Table 3: Band-averaged DMA storage modulus, loss modulus, loss tangent, and Laun-rule-inferred first normal stress difference $\overline{N_1}$ for EPDM-60/70/80 at 80, 100, and 120 °C. Bands are arithmetic means over the full measured frequency range (0.01–100 Hz, 21 logarithmically spaced points per isotherm). The $\overline{N_1}$ ratio between adjacent isotherms supports the Arrhenius cross-validation in Section 2.2.1 of the main text and bounds the wall-to-core thermal-gradient impact on the FMM-corrected swell discussed in Section 3.9.

| **Compound** | $T$ **(°C)** | $\overline{\mathrm{G}'}$ **(Pa)** | $\overline{\mathrm{G}''}$ **(Pa)** | $\overline{\tan\delta}$ | $\overline{N_1}$ **(Pa)** | **n points** |
|---|---|---|---|---|---|---|
| EPDM-60 | 80 | $9.78 \times 10^4$ | $1.77 \times 10^5$ | 2.066 | $5.47 \times 10^5$ | 21 |
| | 100 | $1.14 \times 10^5$ | $1.71 \times 10^5$ | 1.418 | $5.23 \times 10^5$ | 21 |
| | 120 | $6.60 \times 10^4$ | $8.45 \times 10^4$ | 1.173 | $2.61 \times 10^5$ | 21 |
| EPDM-70 | 80 | $9.95 \times 10^4$ | $1.80 \times 10^5$ | 2.039 | $5.53 \times 10^5$ | 21 |
| | 100 | $1.17 \times 10^5$ | $1.77 \times 10^5$ | 1.436 | $5.42 \times 10^5$ | 21 |
| | 120 | $6.72 \times 10^4$ | $8.69 \times 10^4$ | 1.180 | $2.68 \times 10^5$ | 21 |
| EPDM-80 | 80 | $1.02 \times 10^5$ | $1.82 \times 10^5$ | 2.035 | $5.62 \times 10^5$ | 21 |
| | 100 | $1.21 \times 10^5$ | $1.80 \times 10^5$ | 1.415 | $5.51 \times 10^5$ | 21 |
| | 120 | $6.91 \times 10^4$ | $9.06 \times 10^4$ | 1.181 | $2.79 \times 10^5$ | 21 |

**Interpretation.** The $\overline{N_1}$ values at 80 and 100 °C provide the empirical basis for the linear-scaling estimate of the wall-to-core thermal-gradient impact on the FMM-corrected swell discussed in Section 3.9 of the main text. Across the three compounds, the band-averaged $\overline{N_1}$ values at fixed temperature differ by less than 3% at 80 °C, less than 6% at 100 °C, and less than 7% at 120 °C, consistent with the compound-resolved $N_1$ spread reported in Section 3.9. The full per-frequency dataset (189 rows: 3 compounds × 3 temperatures × 21 frequency points) is archived as `polymers-4238061-S3_DMA_data.csv`.
